# Early postoperative enteral nutrition and its impact on postoperative recovery in gastrointestinal tumor surgery

**DOI:** 10.3389/fonc.2025.1649306

**Published:** 2025-10-01

**Authors:** Peng-Hua Weng, Yan Du, Feng Yuan

**Affiliations:** ^1^ Department of General Surgery, Linping Campus, The Second Affiliated Hospital, Zhejiang University School of Medicine, Hangzhou, Zhejiang, China; ^2^ General Surgery Division Three, The First Affiliated Hospital of Soochow University, Suzhou, Jiangsu, China; ^3^ Department of Thoracic Surgery, Zhejiang Hospital, Hangzhou, Zhejiang, China

**Keywords:** gastrointestinal tumors, early postoperative enteral nutrition, surgical site infection, wound healing, nutritional parameters

## Abstract

**Background:**

Surgeries for gastrointestinal tumors frequently result in considerable postoperative complications, such as surgical site infections (SSIs) and impaired nutritional parameters. This study focuses on the role of Early Postoperative Enteral Nutrition (EPEN) in alleviating these difficulties.

**Methods:**

This retrospective study included 110 patients undergoing gastrointestinal tumor surgery between January 2019 and January 2023. Patients receiving EPEN were allocated to the observation group, while others received conventional care. Nutritional indices (total protein [TP], albumin [ALB], and prealbumin [PA]) were measured at baseline and on postoperative day 14. Baseline inflammatory status, including C-reactive protein (CRP), procalcitonin (PCT), white blood cell (WBC) count, and derived ratios such as the neutrophil-to-lymphocyte ratio (NLR), platelet-to-lymphocyte ratio (PLR), and systemic immune-inflammation index (SII), was also assessed. Clinical outcomes included wound healing time and postoperative complications.

**Results:**

EPEN was associated with faster wound healing (10.32 ± 1.32 vs 13.65 ± 0.21 days; P<0.001) and a lower SSI rate (1.82% vs 17.02%; P = 0.015), with no difference in wound bleeding (5.45% vs 9.09%; P = 0.463). At day 14, TP, ALB, and PA were higher in the EPEN group (TP 70.76 ± 4.53 g/L; ALB 39.24 ± 1.87 g/L; PA 297.45 ± 21.32 mg/L; all P<0.001 vs control). Length of stay was similar (7.82 ± 1.21 vs 7.69 ± 1.59 days; P = 0.630). Time to first flatus (33.6 ± 5.8 vs 47.1 ± 7.1 hours; P<0.001), first bowel movement (55.2 ± 6.3 vs 70.5 ± 8.2 hours; P<0.001), and patient satisfaction (8.50 ± 1.10 vs 7.20 ± 1.30; P<0.001) favored EPEN.

**Conclusions:**

In this retrospective study, EPEN might be associated with improvements in nutritional indicators and clinical outcomes, including faster wound healing and reduced surgical site infections in gastrointestinal tumor patients. However, the current data do not provide sufficient evidence to conclusively determine whether the improvements are directly due to EPEN, further prospective, randomized studies are needed to confirm these associations and clarify their causal relationships.

## 1 Introduction

Gastrointestinal (GI) tumors represent a significant public health concern due to their high prevalence and the complexity of their treatment (1, 2). These malignancies, which include a variety of tumors originating in the gastrointestinal tract, are known for their aggressive nature and the substantial morbidity associated with them (3). The treatment modalities for GI tumors often involve surgical intervention, which, while necessary for tumor removal, introduces a range of postoperative challenges. Surgical treatment remains a cornerstone in the management of GI tumors (4). It provides a potential for cure in early-stage tumors and palliation in advanced cases (5). However, the surgical intervention itself is a major physiological stressor, especially in patients already compromised by their nutritional parameters due to the tumor’s effect on the gastrointestinal system (6). This aspect is of paramount importance, as malnutrition is a common and serious issue in patients with GI tumors. Malnutrition not only hampers the recovery process but also predisposes patients to a range of postoperative complications, notably wound infections (7). The relationship between malnutrition and surgical outcomes is well-established in clinical literature (8). Patients with inadequate nutritional parameters face a heightened risk of postoperative complications, including delayed wound healing and increased susceptibility to infections (9). The risk is particularly pronounced in the context of surgeries for GI tumors, where the alimentary tract is directly involved (10). This leads to an important consideration in postoperative care: the optimization of nutritional parameters.

Early postoperative enteral nutrition (EPEN) has emerged as a crucial strategy in this regard. EPEN refers to the administration of nutrients directly into the gastrointestinal tract, typically via a tube, soon after surgery (11). This approach contrasts with the traditional practice of fasting or relying on parenteral nutrition, where nutrients are provided intravenously. EPEN is grounded in the principle that early activation of the gut has multiple benefits, including maintaining the integrity of the gut mucosa, stimulating gut-associated lymphoid tissue, and reducing bacterial translocation, thereby potentially reducing the risk of postoperative infections (12, 13). The potential of EPEN to prevent postoperative wound infections is a topic of significant interest. Wound infection remains a major concern in surgical patients, leading to prolonged hospital stays, increased healthcare costs, and higher morbidity (14). In patients with GI tumors, the risk is compounded by the factors mentioned earlier, making the prevention of such infections a critical component of postoperative care. Furthermore, EPEN plays a crucial role in improving the nutritional parameters of patients’ post-surgery (15, 16). By providing adequate nutrition, EPEN supports the body’s healing processes, improves immune function, and enhances the patient’s overall recovery trajectory. This is particularly beneficial for patients with GI tumors, who are often in a state of preoperative malnutrition or cachexia.

In light of these considerations, the present study aims to rigorously examine the influence of EPEN on postoperative wound infection rates and nutritional parameters in patients undergoing surgery for GI tumors. By focusing on these parameters, the study seeks to contribute valuable insights into the optimization of postoperative care for this patient population, potentially leading to improved clinical outcomes and enhanced quality of life.

## 2 Materials and methods

### 2.1 Study design

A comprehensive retrospective analysis was meticulously conducted at our institution to evaluate the impact of EPEN on surgical site infection and nutritional parameters among patients undergoing surgeries for gastrointestinal tumors. This investigation spanned a period from January 2019 to January 2023. Patients who received EPEN postoperatively were allocated to the observation group for detailed examination. Conversely, patients who did not receive EPEN post-surgery were categorized into the control group. Informed consent was obtained from all subjects. The study was approved by the hospital’s ethics committee and conducted in accordance with relevant guidelines and regulations, including the Declaration of Helsinki. All personal data were anonymized prior to analysis to ensure participant confidentiality and privacy. Perioperative antibiotic prophylaxis was standardized across both groups. All patients received prophylactic broad-spectrum antibiotics—specifically, intravenous cefuroxime (1.5 g every 8 hours)—initiated within 30 minutes before surgical incision and continued for 24 – 48 hours postoperatively. For patients with documented β-lactam allergy, intravenous clindamycin (600 mg every 8 hours) combined with gentamicin (5 mg/kg once daily) was administered.

### 2.2 Inclusion and exclusion criteria

#### 2.2.1 Inclusion criteria

1. Diagnosis: Patients diagnosed with gastrointestinal tumors, confirmed through histopathological examination.
2. Surgical Intervention: Patients who underwent surgical resection for their gastrointestinal tumors.
3. Nutritional Parameters: Patients with varied nutritional parameters at the time of surgery, as assessed by standard nutritional screening tools.
4. Consent: Patients who provided informed consent for participation in the retrospective analysis.

#### 2.2.2 Exclusion criteria

1. Non-GI Tumor Surgery: Patients who underwent surgeries for conditions other than gastrointestinal tumors.
2. Previous Enteral Nutrition: Patients who received enteral nutrition before their surgical procedures.
3. Concurrent Infections: Patients with preoperative infectious conditions, including but not limited to systemic infections or localized infections in the gastrointestinal tract.
4. Chronic Diseases Impacting Nutrition: Patients with chronic diseases that could independently influence nutritional parameters, such as uncontrolled diabetes mellitus, chronic kidney disease, or chronic liver disease.
5. Postoperative Complications: Patients who experienced major postoperative complications unrelated to surgical site infection or nutritional parameters, which could potentially confound the study outcomes (e.g., postoperative hemorrhage, anastomotic leak).

### 2.3 Early postoperative enteral nutrition versus conventional postoperative nutritional approaches

In the EPEN group, patients began receiving enteral nutrition through a gastroenteric feeding tube initiated between postoperative day 1 and day 3. The enteral nutrition formula used was Nuodikang by Nuodixia Pharmaceuticals Ltd., with an energy density of 1.0 kcal/mL. The volume of this formula was incrementally increased, starting at 500 mL (administered at 50 mL/hour, providing 500 kcal and 3 g nitrogen), then progressing to 1,000 mL (at 75 mL/hour, providing 1,000 kcal and 6 g nitrogen), and eventually to 1,500 mL (at 100 mL/hour, providing 1,500 kcal and 12.5 g nitrogen) over the initial days. This regimen was maintained until the 8th postoperative day. From the 4th to the 8th postoperative day, the nutritional products, quantities, and administration methods remained the same as on the third postoperative day. If the target enteral volume was not achieved, the caloric deficit was supplemented with intravenous glucose infusion to ensure adequate total energy provision.

Patients in the non-EPEN group did not receive enteral nutrition through a feeding tube in the early postoperative period. Nutritional support consisted of conventional postoperative care, which typically included delayed initiation of oral intake once gastrointestinal function recovered, and interim reliance on parenteral nutrition when necessary. This standard approach, while commonly practiced, lacks the early gastrointestinal stimulation provided by EPEN and may influence postoperative recovery parameters such as gut motility, wound healing, and nutritional parameters.

### 2.4 Nutritional and clinical surgical measures

In this study, observational indicators were recorded to assess the effects of the interventions on biochemical protein indices, inflammatory status, and surgical outcomes. Serum total protein (TP), albumin (ALB), and prealbumin (PA) were measured at baseline and 14 days after treatment using fasting morning venous blood samples processed with an automated biochemistry analyzer (BC2200, Shenzhen New Industries Biomedical Engineering Co., Ltd.). Inflammatory and immune parameters, including C-reactive protein (CRP), procalcitonin (PCT), white blood cell (WBC) count, neutrophil count, lymphocyte count, and platelet count. Derived indices were calculated as neutrophil-to-lymphocyte ratio (NLR = neutrophils/lymphocytes), platelet-to-lymphocyte ratio (PLR = platelets/lymphocytes), and systemic immune-inflammation index (SII = platelets × neutrophils/lymphocytes). Hematologic parameters were measured using a fully automated hematology analyzer (XN-9000, Sysmex Corporation, Kobe, Japan), and CRP/PCT were determined by immunoturbidimetric and chemiluminescent assays, respectively. Surgical outcomes included the duration of wound healing and the incidence of postoperative SSIs.

### 2.5 Data collection for postoperative recovery and patient tolerance

In this study, key postoperative recovery milestones and patient tolerance were systematically collected. Recovery milestones included the time to first flatus and first bowel movement, which were measured in hours from the time of surgery. Patient satisfaction was assessed using a 10-point Likert scale, where the scores reflected overall satisfaction with postoperative care. Additionally, tolerance to prolonged nasoenteric tube feeding was monitored in the observation group, where enteral nutrition was maintained for eight consecutive postoperative days. Tolerance was evaluated based on patient-reported symptoms, such as discomfort, nausea, and gastrointestinal issues. Significant tube-related complications, including tube dislodgement, blockage, or intolerance, were recorded. Mild discomfort (e.g., nasal or pharyngeal irritation) and nausea were managed by adjusting the feeding regimen as necessary.

### 2.6 Statistical analysis

The statistical evaluation of the data in this study was conducted using the SPSS software, version 27.0. Initially, the data were categorized as either quantitative or categorical. To determine the distribution pattern of each dataset, normality tests were applied. For quantitative data adhering to a normal distribution, the independent sample t-tests were utilized for assessing the statistical significance between groups, and the results were expressed as mean ± standard deviation. In contrast, quantitative data that did not follow a normal distribution were represented using medians and interquartile ranges (M[P25, P75]), and inter-group comparisons were made using the Mann-Whitney U test. Regarding categorical data, these were presented as frequencies and percentages. The Chi-square (χ2) test was employed to examine the independence or associations among these variables. For correlation analysis, Pearson’s correlation coefficient was used for normally distributed quantitative data, Spearman’s rank correlation for non-normally distributed quantitative data, and Kendall’s tau for count data. The testing of hypotheses was bidirectional (two-tailed), and a p-value of less than 0.05 was established as the threshold for determining statistical significance.

## 3 Results

### 3.1 Participant analysis

Baseline demographic and clinical characteristics, inflammatory parameters, tumor types, and surgical methods for both groups are summarized in **Table 1**. In the control group, there were 30 males and 25 females, with an age range of 40 – 61 years and a mean age of 50.54 ± 4.98 years. The distribution of tumor types comprised 23 cases of colon cancer, 17 cases of rectal cancer, 12 cases of gastric cancer, and 3 cases of other gastrointestinal malignancies. Laparoscopic surgery was performed in 39 patients, whereas 16 patients underwent open surgery. In the observation group, 28 males and 27 females were included, aged between 40 and 62 years, with a mean age of 50.79 ± 5.06 years. Tumor types included 22 cases of colon cancer, 18 cases of rectal cancer, 13 cases of gastric cancer, and 2 cases of other gastrointestinal malignancies. Laparoscopic surgery was performed in 36 patients, while 19 patients underwent open surgery. Baseline inflammatory and immune-related parameters were comparable between the two groups. Median CRP levels were 6.2 [3.8 – 10.5] mg/L in the control group and 6.0 [3.5 – 10.0] mg/L in the observation group (P = 0.324). Mean WBC counts were 6.88 ± 1.25 × 10^9^/L and 6.79 ± 1.30 × 10^9^/L, respectively (P = 0.229). No significant differences were observed for neutrophil counts, lymphocyte counts, platelet counts, or derived indices including neutrophil-to-lymphocyte ratio (NLR), platelet-to-lymphocyte ratio (PLR), systemic immune-inflammation index (SII), and procalcitonin (PCT) levels (all P > 0.05).

**Table 1:** Baseline demographic, clinical, inflammatory, and surgical characteristics of patients in the control and observation groups.

Item	Control group (n=55)	Observation group (n=55)	P-value
Gender (Male/Female)	30/25	28/27	0.849
Age Range (years)	40–61	40–62	—
Mean Age (years)	50.54 ± 4.98	50.79 ± 5.06	0.826
CRP (mg/L), median [IQR]	6.2 [3.8 – 10.5]	6.0 [3.5 – 10.0]	0.324
WBC (×10^9^/L), mean ± SD	6.88 ± 1.25	6.79 ± 1.30	0.229
Neutrophils (×10^9^/L), mean ± SD	4.35 ± 1.05	4.29 ± 1.02	0.303
Lymphocytes (×10^9^/L), mean ± SD	1.88 ± 0.42	1.92 ± 0.40	0.593
Platelets (×10^9^/L), mean ± SD	248 ± 52	242 ± 49	0.655
NLR, median [IQR]	2.29 [1.80 – 2.85]	2.23 [1.76 – 2.80]	0.413
PLR, median [IQR]	132 [110 – 155]	126 [108 – 150]	0.406
SII (×10^9^/L²), median [IQR]	561 [420 – 700]	540 [415 – 685]	0.357
PCT (ng/mL), median [IQR]	0.06 [0.04 – 0.09]	0.06 [0.04 – 0.08]	0.143
Tumor Types (cases)			0.962
- Colon Cancer	23	22	
- Rectal Cancer	17	18	
- Gastric Cancer	12	13	
- Others	3	2	
Surgical Methods (cases)			0.682
- Laparoscopic Surgery	39	36	
- Open Surgery	16	19	

### 3.2 Efficacy of EPEN on wound healing time and postoperative complications

The study’s results, encapsulated in **Table 2**, reveal significant disparities between the observation and control groups in terms of postoperative recovery and complication rates. A notable difference was observed in wound healing time, with the observation group showing a considerably shorter duration (10.32 ± 1.32 days) compared to the control group (13.65 ± 0.21 days). This difference was statistically significant, as indicated by a t-value of 18.477 and a p-value of less than 0.001. Furthermore, the incidence of postoperative complications was also found to vary significantly between the two groups. The observation group exhibited a markedly lower incidence of surgical site infection (1.82%) compared to the control group (17.02%), with a Chi-square value of 5.930 and a p-value of 0.015, highlighting a statistically significant difference. However, the incidence of wound bleeding showed no significant statistical difference between the groups, as demonstrated by a Chi-square value of 0.539 and a p-value of 0.463. These findings underscore the effectiveness of the interventions employed in the observation group in enhancing wound healing and reducing the risk of certain postoperative complications, particularly infections.

**Table 2:** Comparison of wound healing time and postoperative complication rates between the two patient groups (n = 55).

Group	Wound healing time (days)	Incidence of surgical site infection [n (%)]	Incidence of wound bleeding [n (%)]
Observation (n=55)	10.32 ± 1.32	1 (1.82%)	3 (5.45%)
Control (n=55)	13.65 ± 0.21	8 (17.02%)	5 (9.09%)
t/χ²	18.477	5.930	0.539
P-value	<0.001	0.015	0.463

### 3.3 Efficacy of EPEN on nutritional parameters in patient groups

The study’s findings, as summarized in **Table 3**, demonstrate significant variations in the nutritional indicators between the observation and control groups, both before and after the treatment. Initially, at the pre-treatment stage, both groups exhibited comparable levels of TP, ALB, and PA, with no significant statistical differences observed between the groups (p-values: TP = 0.850, ALB = 0.482, PA = 0.981). However, a marked improvement in nutritional parameters was observed post-treatment, particularly in the observation group. After 14 days of treatment, the observation group showed a significant increase in all three nutritional indicators: TP increased to 70.76 ± 4.53 g/L, ALB to 39.24 ± 1.87 g/L, and PA to 297.45 ± 21.32 mg/L. These changes were statistically significant compared to their pre-treatment values (p < 0.001 for TP, ALB, and PA). In contrast, while the control group also exhibited an increase in these indicators post-treatment, the improvement was less pronounced and only significant for TP (55.65 ± 5.34 g/L, p < 0.001). The significant post-treatment improvements in TP, ALB, and PA in the observation group as opposed to the control group underline the potential benefits of the treatment strategies employed in the former.

**Table 3:** Comparison of nutritional indicators between patient groups before and after treatment (x ± s, n = 55).

Timepoint	Group	Total protein (TP) [g/L]	Albumin (ALB) [g/L]	Prealbumin (PA) [mg/L]
Pre-treatment	Observation (n=55)	51.65 ± 4.25	33.24 ± 0.87	219.65 ± 24.72
	Control (n=55)	51.81 ± 4.59	33.35 ± 0.76	219.76 ± 24.51
	t-value	0.190	0.706	0.023
	p-value	0.850	0.482	0.981
Post-treatment (14d)	Observation (n=55)	70.76 ± 4.53*	39.24 ± 1.87*	297.45 ± 21.32*
	Control (n=55)	55.65 ± 5.34*	32.95 ± 1.54	223.61 ± 23.54
	t-value	16.002	19.256	17.242
	p-value	<0.001	<0.001	<0.001

*Indicates a statistically significant difference compared to pre-treatment values within the same group (P < 0.05).

### 3.4 Length of stay, postoperative recovery milestones, and patient satisfaction

In terms of hospitalization and postoperative recovery milestones, no statistically significant difference was observed in the mean length of stay between the control group (7.69 ± 1.59 days) and the observation group (7.82 ± 1.21 days; P = 0.630). The observation group demonstrated a shorter mean time to first flatus (33.6 ± 5.8 hours) compared with the control group (47.1 ± 7.1 hours; P < 0.001). Similarly, the mean time to first bowel movement was shorter in the observation group (55.2 ± 6.3 hours) than in the control group (70.5 ± 8.2 hours; P < 0.001). The patient satisfaction score was higher in the observation group (8.50 ± 1.10) than in the control group (7.20 ± 1.30; P < 0.001) (**Table 4**).

**Table 4:** Comparison of length of stay, recovery milestones, and patient satisfaction between the two groups.

Outcome measure	Control group (n=55)	Observation group (n=55)	P-value
Length of Stay (days)	7.69 ± 1.59	7.82 ± 1.21	0.630
First Flatus (hours)	47.1 ± 7.1	33.6 ± 5.8	< 0.001
First Bowel Movement (hours)	70.5 ± 8.2	55.2 ± 6.3	< 0.001
Patient Satisfaction (1 – 10 scale)	7.20 ± 1.30	8.50 ± 1.10	< 0.001

### 3.5 Tolerance to prolonged nasoenteric tube feeding

In the EPEN group, nasoenteric tube feeding was maintained for eight consecutive postoperative days. Tolerance was generally high, with no patients discontinuing enteral nutrition prematurely due to tube-related intolerance. Mild discomfort, such as transient nasal or pharyngeal irritation, occurred in 7 patients (12.7%) and resolved spontaneously without intervention. Minor nausea was observed in 2 patients (3.6%) during the early feeding period and was managed by temporarily reducing the infusion rate. No cases of tube dislodgement, blockage, or significant gastrointestinal intolerance (e.g., persistent vomiting, severe diarrhea) were recorded. All patients in the EPEN group completed the planned enteral nutrition regimen via the feeding tube.

## 4 Discussion

The interplay between nutritional parameters and surgical outcomes in patients undergoing surgery for gastrointestinal tumors is a complex and critical aspect of postoperative care. The influence of EPEN on surgical site infection and nutritional parameters, as investigated in our study, is grounded in the growing recognition of nutrition as a pivotal factor in patient recovery (17, 18). Gastrointestinal surgeries, by their nature, pose significant challenges, including disruptions to the normal function of the digestive system and an increased risk of postoperative complications, notably surgical site infections (SSIs) (19, 20). Historically, the standard postoperative protocol often involved delayed initiation of feeding, based on the belief that gastrointestinal rest was necessary for healing. However, this approach may inadvertently contribute to malnutrition, compromised immunity, and delayed wound healing. Recent paradigms in surgical care have shifted towards the early introduction of enteral nutrition, recognizing its role in maintaining gut integrity, modulating immune responses, and promoting faster recovery. EPEN targets the critical period immediately following surgery when the risk of infection is highest, and the body’s need for nutrients to fuel the healing process is substantial. By administering nutrition directly to the gut, EPEN not only provides essential nourishment but also stimulates the gut-associated lymphoid tissue, an integral component of the immune system (21). This dual benefit of nutritional support and immune modulation by EPEN could be pivotal in reducing SSIs, a major concern in postoperative care for patients with gastrointestinal tumors.

Our study’s results shed light on the significant impact of EPEN on postoperative recovery in patients with gastrointestinal tumors, highlighting faster wound healing and reduced complication rates in the observation group compared to the control group. The underlying mechanisms of these observed benefits appear to be deeply intertwined with the physiological responses to surgery and the role of nutrition in postoperative care. The notable improvement in wound healing times in the observation group receiving EPEN can be attributed to the direct nutritional support provided to the gastrointestinal tract (22). Enteral nutrition, unlike parenteral nutrition or delayed oral feeding, offers a unique advantage by directly nourishing the gut, which is essential for maintaining its integrity and function. This approach likely contributes to preserving the gut mucosal barrier, thereby reducing the risk of bacterial translocation and subsequent infections that are common postoperative complications (11). Moreover, the presence of adequate nutrients from EPEN supports cellular metabolism crucial for tissue repair and healing, particularly in the synthesis of collagen and other structural proteins.

Furthermore, the enhanced nutritional parameters reflected by increased levels of Total Protein, Albumin, and Prealbumin in the observation group underscores the effectiveness of EPEN in meeting the elevated metabolic demands following surgery (23). Surgery typically induces a catabolic state in the body, characterized by muscle breakdown and increased energy requirements (24). EPEN helps counteract this state by providing essential nutrients, thereby preventing excessive muscle loss and supporting overall protein status (25). The improvement in these nutritional parameters is not just a reflection of nutrient intake but also indicates enhanced protein synthesis, particularly in the liver (26). This is critical for recovery, as proteins like Albumin and Prealbumin are key markers of nutritional parameters and play vital roles in maintaining plasma osmotic pressure and transporting various substances in the blood. In contrast, the control group, which relied on traditional postoperative nutritional approaches, showed less pronounced improvements. This could be due to the delayed initiation of nutritional support post-surgery, which potentially prolonged the catabolic state and adversely affected wound healing and recovery (27). Additionally, the traditional methods of nutrition may not provide nutrients in the most physiologically beneficial manner, especially concerning gut health and immune function.

The use of visceral proteins such as TP, ALB, and PA as nutritional markers in postoperative patients can be confounded by acute inflammation, which is a well-known limitation in surgical settings. These proteins are often classified as acute-phase reactants and may reflect the inflammatory response rather than an accurate assessment of nutritional parameters. In our study, however, we aimed to minimize these confounding effects to ensure that the observed differences in nutritional markers between the groups were primarily due to the EPEN intervention, rather than surgical factors or acute-phase responses. To address this, we employed several strategies to strengthen the validity of our findings. First, baseline comparability was ensured between the two groups, as the distribution of surgical approaches (laparoscopic vs. open surgery) was similar, reducing the risk of confounding by surgical method. Second, a stratified analysis was performed by surgical approach, and the beneficial effects of EPEN on wound healing time and surgical site infection rates were consistent across both laparoscopic and open surgery subgroups. This suggests that the observed benefits were not influenced by the type of surgery performed. Additionally, to minimize the potential impact of acute inflammation on the nutritional markers, we measured TP, ALB, and PA at baseline (preoperatively) and 14 days post-treatment, rather than in the immediate postoperative period. By this time, the acute inflammatory response typically subsides. Moreover, pre-treatment levels of TP, ALB, and PA were comparable between the two groups, ensuring that the observed changes could not be attributed to baseline differences in inflammatory responses. Finally, the improvements in nutritional markers were closely aligned with clinical outcomes, including shorter wound healing times and lower rates of SSIs in the EPEN group. This consistency between biochemical and clinical results further supports the conclusion that the observed improvements were reflective of true nutritional benefits, rather than the resolution of inflammation. In summary, our study demonstrates that early enteral nutrition can significantly improve both nutritional markers and clinical outcomes, such as wound healing and infection rates, though further research is needed to confirm the role of nutrition in these improvements.

Regarding the influence of acute-phase responses on visceral protein levels, additional inflammatory and immune parameters were collected and analyzed to assess baseline inflammatory status. These included CRP, PCT, white blood cell count, neutrophil and lymphocyte counts, platelet count, and derived indices such as NLR, PLR, and the SII. All parameters were measured within 24 hours before surgery. Comparative analysis revealed no statistically significant differences between the control and observation groups for any of these markers (all P > 0.05), indicating that the two cohorts had comparable baseline inflammatory status. This baseline equivalence reduces the likelihood that differences in postoperative visceral protein indices were attributable to pre-existing disparities in inflammatory activity. While postoperative inflammatory marker trends were not available, the alignment between improved protein indices and favorable clinical outcomes in the EPEN group supports the plausibility that the observed changes are at least partly nutrition-driven rather than solely reflective of inflammation resolution. Nonetheless, we acknowledge that integrating serial postoperative inflammatory marker measurements (e.g., IL-6, TNF-α) and functional nutritional assessments in future prospective studies would strengthen causal inference.

Several limitations should be acknowledged. First, the retrospective nature of the study may introduce biases related to data collection and patient selection. Retrospective analyses often rely on existing records, which might not contain all pertinent variables or detailed patient information. Second, the study’s patient population was drawn from a single institution, which may limit the generalizability of the findings to broader, more diverse populations. Finally, the lack of randomization in group allocation could potentially lead to confounding factors influencing the outcomes. These limitations highlight the need for prospective, randomized controlled trials to further validate and expand upon our findings.

## 5 Conclusions

In this retrospective study, the use of EPEN might be associated with improvements in nutritional indicators, such as total protein, albumin, and prealbumin, as well as enhanced clinical outcomes, including shorter wound healing times and reduced surgical site infection rates in patients undergoing gastrointestinal tumor surgery. However, the current data do not provide sufficient evidence to conclusively determine whether the improvements are directly due to EPEN, as factors such as inflammation could also influence these outcomes. Further prospective, randomized studies with additional data on inflammation levels beyond the immediate postoperative period are needed to more thoroughly assess the causal relationships between EPEN and nutritional recovery.

## Data Availability

The raw data supporting the conclusions of this article will be made available by the authors, without undue reservation.
